# Exploring dietitians' salient beliefs about shared decision-making behaviors

**DOI:** 10.1186/1748-5908-6-57

**Published:** 2011-06-01

**Authors:** Sophie Desroches, Annie Lapointe, Sarah-Maude Deschênes, Marie-Pierre Gagnon, France Légaré

**Affiliations:** 1 CHUQ Research Center, Centre Hospitalier Universitaire de Québec-Hôpital St-François-d'Assise, Québec, QC, Canada; 2 Department of Food and Nutrition Sciences, Laval University, Québec, QC, Canada; 3 Faculty of Nursing, Laval University, Québec, QC, Canada; 4 Department of Family and Emergency Medicine, Laval University, Québec, QC, Canada

## Abstract

**Background:**

Shared decision making (SDM), a process by which health professionals and patients go through the decision-making process together to agree on treatment, is a promising strategy for promoting diet-related decisions that are informed and value based and to which patients adhere well. The objective of the present study was to identify dietitians' salient beliefs regarding their exercise of two behaviors during the clinical encounter, both of which have been deemed essential for SDM to take place: (1) presenting patients with all dietary treatment options for a given health condition and (2) helping patients clarify their values and preferences regarding the options.

**Methods:**

Twenty-one dietitians were allocated to four focus groups. Facilitators conducted the focus groups using a semistructured interview guide based on the Theory of Planned Behavior. Discussions were audiotaped, transcribed verbatim, coded, and analyzed with NVivo8 (QSR International, Cambridge, MA) software.

**Results:**

Most participants stated that better patient adherence to treatment was an advantage of adopting the two SDM behaviors. Dietitians identified patients, physicians, and the multidisciplinary team as normative referents who would approve or disapprove of their adoption of the SDM behaviors. The most often reported barriers and facilitators for the behaviors concerned patients' characteristics, patients' clinical situation, and time.

**Conclusions:**

The implementation of SDM in nutrition clinical practice can be guided by addressing dietitians' salient beliefs. Identifying these beliefs also provides the theoretical framework needed for developing a quantitative survey questionnaire to further study the determinants of dietitians' adoption of SDM behaviors.

## Background

The past two decades have witnessed growing interest in the decision-making processes that occur during clinical encounters. One of these processes is shared decision making (SDM), in which a healthcare choice is made jointly by the health professional and the patient [1]. SDM is primarily employed in cases where several treatment alternatives are available, but there is no single best option. Examples include treatments for type 2 diabetes [2] and hypertension [3]. SDM is positioned as the middle ground between the paternalistic model, where the health professional assumes the leading role in treatment decisions, and the informed patient choice model, where the health professional's role is limited to giving information and the patient is responsible for deciding on treatment [4,5].

SDM is increasingly advocated in healthcare because of its potential to improve the decision-making process for patients and increase patients' adherence to the treatment decision, improving patient outcomes as a result [6,7]. SDM is also one of the core features of patient-centered care [8] and is increasingly intertwined with evidence-based practice [9]. Despite growing clinical interest in SDM, barriers to its implementation remain [10], and SDM has not yet been widely adopted by health professionals [11]. This said, SDM comprises a set of behaviors that could be modified by activities designed to foster its practice. According to a systematic review by Makoul and Clayman, the two elements most frequently considered by the literature to define SDM are, first, the health professional's presentation of treatment options to the patient and, second, the health professional's clarification of the patient's values and preferences [12]. Studies show that physicians find these two behaviors difficult to perform [13]. Less is known about whether other health professionals, such as dietitians, encounter the same difficulty, the vast majority of studies on SDM having been conducted among patients [6] and physicians [10].

The increase in the number of evidence-based dietary options recommended to prevent and manage risk factors associated with diet-related conditions such as obesity [14-16] and cardiovascular diseases [17-19] represents an opportunity to better individualize dietary treatments to match patients' preferences, values, and lifestyles. Concurrently, through television, newspapers, and magazines, and more recently cyberspace, patients are increasingly exposed to nutritional information whose accuracy varies [20,21]. As a result, patients facing diet-related decisions are more able than ever to participate actively in their own dietary care, but at the same time, may feel overwhelmed by the volume of information at their disposal. This puts patients at risk of making poor dietary decisions [21,22]. In this context [23], SDM's promotion of clinical practices that are evidence based and patient centered hold great promise for increasing dietary treatment decisions that are informed and grounded in patients' values.

### Conceptual framework

Researchers have used social cognitive theories to improve our understanding of a variety of health-related behaviors, including those of health professionals [24]. Most SDM models refer to a set of competencies or behaviors [1,12] in which the health professional and the patient must engage in order for SDM to take place. But we lack sufficient knowledge about the psychosocial determinants underlying the adoption or nonadoption of SDM behaviors by patients and health professionals [25,26].

**Figure 1:**
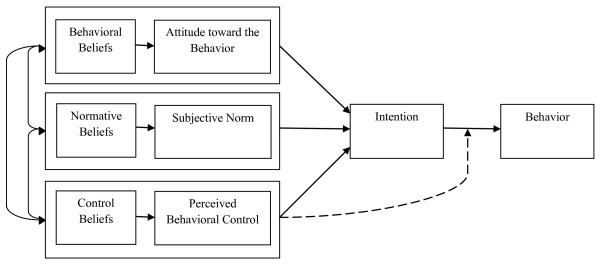
**Ajzen's Theory of Planned Behavior**[27].

The Theory of Planned Behavior (TPB) (Figure 1) [27] suggests that there are three primary determinants of a party's intention to perform a given behavior: (1) the party's attitudes toward performing the behavior, (2) the party's subjective norms with respect to performing the behavior, and (3) the party's perceived behavioral control (*i.e.*, whether the party perceives himself or herself as being able to perform the behavior). Each of these primary constructs is the function of underlying salient beliefs. Attitudes reflect behavioral beliefs about whether engaging in the behavior will produce favorable outcomes; perceived subjective norms reflect normative beliefs about the social pressure to engage or not to engage in the behavior; and perceived behavioral control reflects beliefs, shaped by the party's experience, about his/her ability to adopt a particular behavior. A recent systematic review indicates that the measure of intention is a valid proxy for health professionals' behavior [28] and that the TPB is the theory most frequently used with health professionals [24]. To the best of our knowledge, only two studies have used the TPB to identify the determinants of dietitians' behavioral intentions [29,30].

The objective of the present qualitative study was therefore to identify dietitians' salient beliefs regarding their adoption of the two SDM behaviors most frequently used by the literature to define SDM [12]. One of the behaviors relates to evidence-based practice, while the other relates to patient-centered care. In the context of individual clinical encounters with patients in a hospital setting, we defined the two behaviors as follows: (1) the dietitian presenting the evidence-based dietary treatment options for a given health condition (including the option of doing nothing) to the patient and (2) the dietitian helping the patient clarify his/her values--what was most important to him/her--regarding the options presented.

## Methods

### Participants and recruitment

Dietitians having inpatient or outpatient clinical activities were recruited from hospitals located in the Quebec City metropolitan area. The inclusion criterion for participating in the study was membership in the Professional Order of Dietitians of Quebec, Quebec's dietitians' professional regulatory body. Prior to starting the study, one of the investigators (SD) met dietitians during one of their weekly meetings at their workplace to request their participation in the study once ethical approval was obtained. During the meeting, SD informed dietitians of the objectives of the study and the time commitment that participating in the study would entail. After the Research Ethics Board of the Centre Hospitalier Universitaire de Québec granted the study ethical approval, the team worked with the three clinical nutrition coordinators to schedule dates for focus groups (see below). The coordinators then communicated with the dietitians eligible to participate in the study, inviting them to take part. Participants received no honorarium. All participants gave written informed consent.

The list of participants' names was kept confidential; names were known only to the principal investigator, the project coordinator, and other participants in the same group. Participants' responses were considered as being group responses and were not linked to individual respondents.

### Data collection procedures

Of the 40 dietitians eligible to participate in our study, 21 volunteered to participate and 19 declined. We did not gather information about those who declined. Between January and April 2009, we integrated four focus groups into the weekly meetings of the dietitians. We held two focus groups in the same working site; this allowed us to accommodate a greater number of participants. Groups ranged from three to seven participants, and discussions lasted between 38 and 72 minutes. Each focus group began with one of the investigators (SD) making a 15-minute didactic presentation in which she introduced the concept of SDM and discussed behaviors deemed essential to engage in SDM. Because SDM was a new approach for the dietitians, SD's presentation focused on describing the two behaviors being studied (presenting options and clarifying values) in the context of dietitians' clinical practice. In this way, we sought to ensure that participants would respond to our questions in light of these two behaviors and not others. After the presentation, a trained research coordinator working for one of the investigators (MPG) led a focus group through 12 standardized, semistructured, open-ended questions (six for each behavior). These questions were based on the TPB and were prepared ahead of time. The questions assessed dietitians' behavioral beliefs (what they saw as the advantages and disadvantages of the behaviors), normative beliefs (whether they thought that people important to them would approve or disapprove of the behaviors), and control beliefs (what they considered barriers and facilitators to their practice of the behaviors). The two behaviors were as follows: (1) presenting the evidence-based dietary treatment options for a given health condition (including the option of doing nothing) during the dietitian-patient encounter and (2) helping patients clarify their values or what was most important to them concerning the evidence-based dietary treatment options, again during the dietitian-patient encounter. To avoid confusion between the two behaviors, participants were invited to take a 15-minute break after answering questions related to behavior 1 and before answering questions related to behavior 2. During the break, refreshments were served and participants filled out an anonymous questionnaire assessing their satisfaction with the project thus far.

The focus group discussions were audiotaped and transcribed verbatim for analysis. Transcripts were checked for accuracy, and a copy of the original audio recording, as well as field notes, was kept available for reference during the analysis.

### Data analysis

Two individuals (SMD, AL) independently performed thematic content analysis of the focus group discussions following the elicitation study methodology proposed by Francis *et al*. [31] and Godin and Gagné [32]. The two assessors familiarized themselves with the data by reading the transcripts prior to analysis. They then used NVivo software (version 8, QSR International, Cambridge, MA) to organize the quotes according to a basic set of codes that reflected three TPB-based categories of beliefs: behavioral beliefs, normative beliefs, and control beliefs (Figure 1). Within each belief category, the assessors aggregated similar response items into themes. The assessors then compared their themes to reach consensus over the terminology to be used for each. Most of the time, this exercise led them to reword the names of the themes. On a few occasions, they eliminated themes and reassigned items to a broader theme. A third investigator (SD) was available to resolve any discrepancies. The assessors recorded the number of quotations for each theme and noted the focus group from which each quotation originated. To determine the point of saturation, they calculated the extent to which different focus groups mentioned the same themes and found that, by the end of the third focus group, 93% of themes had been mentioned at least once; the remaining 7% of themes were only mentioned in the fourth focus group. We analyzed the data in the original French transcript--the quotations in Tables 1 and 2 have been translated from French into English.

**Table 1:** Salient beliefs associated with presenting evidence-based dietary treatment options (including the option of doing nothing) during the clinical encounter

Salient beliefs	Quotes illustrating the belief	**Frequency of mention**^**a**^
**Behavioral beliefs--perceived advantages**		

Improves the patient's adherence to treatment	"Involving the child, even if he is young, in the choice: 'What do you want to try between this and that?' (...) If the child chooses on his own, he is more likely to stick to the treatment."	4

Allows the patient to make an informed choice	"An informed decision is when he [the patient] knows them all, all the possible options. So it is really more informed, several options are being offered."	4

Gives control to the patient	"It is not just the health professional who controls the disease, it is also the patient himself."	2

Gives the patient a sense of responsibility	"I think it would give a sense of responsibility to the patient."	2

**Behavioral beliefs--perceived disadvantages**		

Increases the patient's insecurity	"...it could confuse him [the patient] in his decision and then he [the patient] wouldn't know what to do anymore."	3

Increases the dietitian's feeling of incompetence	"I don't know, maybe that presenting all the options could make some patients see us [dietitians] as being less expert (...) because there are some [patients] who like to come here and have the dietitian say, 'Here is where we are going,' whereas now we seem to present a lot of things and finally, they decide for themselves..."	3

**Normative beliefs--approval**		

Physician	"The physician who takes the time to explain the diagnosis..."	3

Multidisciplinary team	"I would say the multidisciplinary team. Often, we will come to the same conclusions."	3

Patient	"The patient, for sure."	2

Patient's family	"The husband, the wife, mostly if it is the wife who is responsible for it all [food preparation] so..."	3

**Normative beliefs--disapproval**		

Physician	"It depends on the attitude, some physicians are more authoritative and they'd rather that we [dietitians] say what they told us."	3

Multidisciplinary team	"Yes, it's true that it could not be well perceived by the team, at first, if the person didn't want to do anything and we didn't help her..."	2

Dietitian	"I would never offer that option [to do nothing]."	2

Patient's family	"There are families, sometimes, who don't like us to provide several [treatment] options."	2

**Control beliefs--barriers**		

Patient's medical condition	"In my area of practice, yes, sometimes, there may be choices to propose but sometimes, there is no choice. A disease has to be treated and the patient's life depends on it [the treatment] so there is no choice, treatment is imposed. In these cases, it`s not possible to engage in shared decision making."	4

Lack of time	"Time. When we want to be quick, sometimes it's better to go right to recommendations."	4

Unmotivated patient	"Maybe the level of motivation. Sometimes, when they [the patients] are not really motivated, you cannot...scare them at first, so targeting only one treatment..."	4

Poor social/familial environment	"Another barrier for us [dietitians] is not having the family's support, the support of the husband, the support of the wife."	3

Patient's personality	"It's all a matter of personality, I think. Some [patients] are annoyed at being presented with [treatment options], and we feel like we're wasting our time."	3

Patient's understanding	"You present all the options, but does the patient understand all the implications..."	3

Disapprobation by the physician	"If the physician doesn't believe in the treatment that you want to use with the patient, he [the physician] won't support you..."	2

Hospital context	"You know, here [at the hospital] is not the place for it. They [the patients] are in a bed; they are looking forward to leaving. They are more than one to a room."	3

Dietitian's professional ethics	"For me, it's about professional ethics."	2

**Control beliefs--facilitators**		

Availability of time	"It's easier with patients whom you've seen in several clinical encounters."	4

Good social/familial environment	"Having a good financial situation, not living in an institution, having the choice to...having control over their [patients' own] lives."	3

Discussions with multidisciplinary team	"We can meet and discuss cases. Because we can say: I came to this conclusion, we took this decision, we can change and..."	2

Motivated patient	"The interest of the patient, his or her openness and receptivity to information."	2

Patient's medical condition	"...who [patients] have chronic diseases, it's less acute..."	2

Support by the multidisciplinary team	"So the multidisciplinary team must also be part of the process..."	2

Increased workforce in clinical nutrition	"If our workload were decreased, or if they (the human resources department) increased the workforce [in clinical nutrition] ..."	2

^a^"Frequency of mention" refers to the number of focus groups, out of a total of four, in which the theme was mentioned.

**Table 2:** Salient beliefs associated with helping patients clarify their values and preferences regarding evidence-based dietary treatment options

Salient beliefs	Quotes illustrating the belief	**Frequency of mention**^**a**^
**Behavioral beliefs--perceived advantages**		

Targets the treatment	"If their [patients'] values include having fun, going to restaurants, sharing meals, this is important so we consider these values in our options."	4

Improves the patient's adherence to the treatment	"Adherence to treatment, again."	4

Increases the patient's trust in the dietitian	"Maybe it establishes a sense of respect between the health professional and the patient if the patient sees that the dietitian respects his values."	4

Increases the patient's awareness	"Making him [the patient] conscious about his values. For some it's unconscious, they do it but they don't realize it."	2

Increases the patient's satisfaction	"His [the patient's] satisfaction. Feeling a bit more involved, like we don't decide for him, he gets the feeling that he's not just a number in this big healthcare system, he's directly involved. So there is probably some kind of appreciation for this approach."	2

Saves time	"It is the opposite of presenting all the options, which requires more time, but when you know your patient's values and preferences, maybe you can save time and not spend an hour with the patient ..."	2

**Behavioral beliefs--perceived disadvantages**		

Confronts the patient	"There are some [patients] who don't like being confronted."	2

**Normative beliefs--approval**		

Patient's family	"The family."	3

Multidisciplinary team	"Multidisciplinary teams, with nurses, physicians..."	4

Physician	"Physicians."	2

**Normative beliefs--disapproval**		

Patient's family	"... so if you try to deconstruct some values that were transmitted by the family... In my opinion, it's the only people [family] who I see who might find it inconvenient."	2

Multidisciplinary team	"It all depends on who is involved, what team."	2

**Control beliefs--barriers**		

Lack of time	"We don't have time to question the patient. It's possible that we don't delve into his values."	4

Lack of patient openness	"He [the patient] may not be interested in opening up to each health professional..."	4

Patient's medical condition	"The fact also that sometimes, in some departments, for example if I think about surgery, when we see surgery patients, it's not when they're at their best."	4

Patient's age	"...when it's been many years that you [the patient] have adopted a behavior, it's always more difficult to question and discuss it [the behavior]."	2

Patient has little trust in the dietitian	"...if we are not able to establish trust right from the beginning, we can't go very far."	2

Patient does not express him/herself clearly	"...or a patient that is not able to express himself very clearly."	2

**Control beliefs--facilitators**		

Patient trusts the dietitian	"To establish trust [with the patient]."	2

Dietitian has enough time	"Again, to be able to follow up with the patient."	3

Patient's family support	"When the entire family is willing to change their behavior, the children, the spouse make the changes too and everyone is motivated."	2

Motivated patient	"If the decision comes from the patient, that's another facilitator."	2

Good listening ability on the part of the dietitian	"If you [the dietitian] understand why he [the patient] has difficulty managing his body weight: because he has an overloaded work schedule, if you listen to him..., then you facilitate the process."	2

Good openness on the part of the patient	"Patient openness."	2

^a^"Frequency of mention" refers to the number of focus groups, out of a total of four, in which the theme was mentioned.

## Results

All participants were female dietitians between 24 and 60 years of age (mean age was 39.3 ± 11.3 years). Their mean number of years in practice was 13.2 ± 9.4 years (range 2 to 29 years). All participants worked in a hospital: 44% only saw inpatients, 37% only saw outpatients, and 19% saw a mix of both. Seventy-five percent were employed full time, and 25% were employed part time. Their clientele varied greatly and included type 1 diabetics, type 2 diabetics, patients undergoing surgery, oncology patients, women having a high-risk pregnancy, patients with a cardiovascular disease, and patients with an eating disorder.

The results that follow are grouped by behavior and theoretical category, as organized in the focus group interview guide. Themes that were mentioned in two or more focus groups were assigned the theoretical category of salient beliefs. Quotations that illustrate each theme within the three theoretical categories are given in Tables 1 and 2.

### Salient beliefs

#### Behavior 1: presenting evidence-based dietary treatment options during the dietitian-patient clinical encounter

As shown in Table 1, every focus group mentioned improving patients' adherence to treatment as an advantage of presenting evidence-based dietary treatment options (including the option of doing nothing) during the clinical encounter. Participants also discussed their perceptions of the disadvantages of presenting the options to patients; these included making patients feel less secure and increasing dietitians' feelings of incompetence.

As regards important people who might approve of dietitians presenting evidence-based dietary treatment options to patients, participants mentioned the multidisciplinary team, the patient's family, and the physician. In three out of four focus groups, they identified physicians as important people who might disapprove.

Barriers associated with presenting the evidence-based dietary treatment options included the patient's medical condition, the lack of time for the dietitian to interact with the patient, an unmotivated patient, a patient's poor social/familial environment, the patient's personality, the patient's understanding, and the hospital milieu.

#### Behavior 2: helping patients clarify their values and preferences regarding dietary treatment options

As shown in Table 2, many dietitians perceived the following advantages to their helping patients clarify their values regarding evidence-based dietary treatment options: it would allow them to target the patient's treatment more precisely, it would improve the patient's adherence to the treatment, and it would reinforce the patient's trust in the dietitian.

With regard to normative beliefs, the multidisciplinary team and the patient's family were mentioned as people who might approve the behavior.

The barrier to the clarification of patients' values regarding their dietary options most often cited by dietitians was the dietitian's lack of time. Some of the other control beliefs explaining barriers and mentioned by dietitians involved the patient: the patient's lack of openness and the patient's medical condition. Having more time to meet patients and having more time to explore the patient's thoughts were both identified as important conditions for the dietitian's ability to clarify the patient's values.

## Discussion

This study is the first to use the TPB to identify dietitians' salient beliefs regarding the adoption of two SDM behaviors. It addresses several gaps in the research on SDM. First, it expands the prospects of implementing SDM beyond the medical profession by providing insight into dietitians' salient beliefs regarding SDM. Second, by reporting on two behaviors corresponding to key concepts of the SDM process--namely, evidence-based practice and patient-centered care--it generates a knowledge base for the design of future theory-based interventions that aim to foster the implementation of SDM in clinical practice.

Several of the advantages that our respondents associated with the SDM behaviors studied here are consistent with previously reported benefits of SDM interventions, such as improving patients' adherence to treatment and increasing patients' satisfaction [6,33]. Reporting on these outcomes in future studies of the effectiveness of SDM in nutritional interventions could thus make SDM more valuable to dietitians and facilitate its uptake and implementation.

With respect to normative beliefs, dietitians frequently mentioned patients or patients' families, physicians, and multidisciplinary teams as important parties who might approve or disapprove of the two behaviors of study. This suggests that when seeking to identify the determinants of patients' involvement in decision making, future SDM studies should consider these parties' roles. It also suggests that there would be merit to developing interventions for enhancing an interprofessional approach to SDM that would foster a common understanding of SDM among health professionals at the point of care [34].

As stated earlier, even though the clinical community is demonstrating growing interest in SDM, many barriers to the implementation of SDM remain, and health professionals have yet to adopt the approach widely [11]. The control beliefs identified by dietitians in our study are congruent with a systematic review of 38 studies on the barriers and facilitators to implementing SDM in clinical practice as perceived by health professionals [10]. Interestingly, although 89% of the health professionals covered in the 38 studies of the systematic review were physicians [10], many of the barriers they cited were similar to those cited by dietitians. These included time constraints, SDM's lack of applicability due to the patient's characteristics, and SDM's lack of applicability due to the patient's clinical situation. This suggests that at least to some extent, a cohesive set of determinants may underlie the exercise of SDM behaviors across health professions. It is also worth noting that dietitians identified several barriers that were related to patients, such as patients' motivation, their comprehension, their personality, and their health condition. This raises the concern that rather than practicing SDM with those patients who, if afforded the opportunity, would choose to take part in nutrition-related decisions, dietitians might only practice SDM with patients whom they think would be good candidates for SDM, in other words, patients whom dietitians had screened. One remedy would be to study the factors influencing patients' preferences of involvement in nutrition-related decision making, in a bid to preempt dietitians' assumptions in this regard. Any such study would, of course, have to account for the evolution of patients' preferences in SDM; these preferences appear to be variable and to change over time, depending on a number of factors [35].

### Strengths and limitations

Focus groups produce data of high quality and are important tools in health research,[36] and our use of focus groups constitutes an important strength of our study. Another strength of our study was our use of the TPB to assess participants' salient beliefs regarding the exercise of two SDM behaviors. Very few studies have used a theory-based approach to predict the determinants of behaviors essential for SDM to take place, and no such studies have been conducted with dietitians [37,38]. This has considerably limited the development of interventions to facilitate the implementation of SDM in practice, since theories and models are essential for a systematic analysis of the factors influencing the use of evidence in clinical, organizational, and policy decisions [39]. In addition, our study is the first to uncover beliefs underlying dietitians' attitudes, subjective norms, and perceptions of control with regard to a patient-centered behavior (SDM behavior 2). It is also the first to offer a theory-based categorization of determinants in line with patient-centered care. This categorization will facilitate the elaboration of educational activities that target barriers--identified by dietitians themselves--that fall within the TPB construct of perceived behavioral control.

Our study also has limitations. The participants in our study were dietitians practicing in a hospital setting within a single Canadian province; Canada's healthcare system is actually a collection of provincial, territorial, and (in a few small cases) federal healthcare systems whose hospital and nonhospital settings have similarities but also differ. Therefore, we cannot extrapolate all of the salient beliefs identified in our focus groups to other populations. Furthermore, we have no data on those dietitians who declined to participate in our study. Given the broad ranges of age, experience, and expertise of dietitians who participated, however, we consider our sample to be representative.

A possible limitation of our study is that it may have introduced a social desirability response bias, whereby participants gave socially acceptable responses rather than their actual opinions or answers that reflected real practice. In an effort to minimize desirability bias, we arranged to have focus group discussions led by a researcher with expertise in social cognitive theories but without an academic background related to nutrition or dietetics. Another way to minimize the desirability effect would have been to offer dietitians individual interviews. Our decision to conduct focus groups rather than individual interviews stemmed mainly from our need to facilitate recruitment and reduce participants' time commitments: we scheduled the focus groups during times when dietitians were already available for their weekly group meeting with their colleagues and coordinator.

Another potential limitation of our study is that it is based on SDM research that, although current, might not have captured every step of the SDM process [12]. To some extent, we controlled for this limitation by targeting more than one behavior (in this, our study is the first of its kind with health professionals). To remedy this limitation fully, however, we would have had to develop a questionnaire comprising all conceivable SDM behaviors [12]. However ideal from a conceptual viewpoint, such a questionnaire would have been burdensome for study participants, and its length could have worsened the quality of their responses. It could also be argued that this article could have discussed the two behaviors without reference to SDM. However, too often evidence-based practice is perceived as excluding patients' perspectives, and patient-centered care is studied without considering the importance of evidence-based practice. SDM represents a way to level these silos; it is the ideal model in the sense that it recognizes the interdependence of the two behaviors and calls upon practitioners to use them together to improve the quality of healthcare. For that reason, we preferred to discuss them together.

## Conclusions

This study is the literature's first attempt to construct a theoretical basis for guiding the implementation of SDM in nutrition clinical practice. Researchers can draw on dietitians' salient beliefs as identified here to develop a quantitative questionnaire that elucidates dietitians' intentions to adopt the two behaviors deemed essential for SDM to occur and to clarify the psychosocial determinants of those intentions. SDM represents a fundamental change in health professionals' clinical practices, and a better understanding of dietitians' positions vis-à-vis SDM is essential to teaching dietitians to share nutrition-related decisions with their patients when more than one treatment option is available. The benefits of dietitians' involvement in SDM have yet to be quantified, but the promise for patient outcomes is great.

## Competing interests

The authors declare that they have no competing interests.

## Authors' contributions

SD conceived of and designed the study, analyzed and interpreted the data, and wrote the manuscript. AL analyzed and interpreted the data, helped draft the manuscript, and revised the manuscript. SMD analyzed and interpreted the data and revised the manuscript. MPG and FL conceived of and designed the study and revised the manuscript. All authors read and approved the final manuscript.
